# Pre-existing *Toxoplasma gondii* infection increases susceptibility to pentylenetetrazol-induced seizures independent of traumatic brain injury in mice

**DOI:** 10.3389/fnmol.2022.1079097

**Published:** 2023-01-05

**Authors:** Tamara L. Baker, Alessandro D. Uboldi, Christopher J. Tonkin, David K. Wright, Anh Vo, Trevor Wilson, Richelle Mychasiuk, Stuart J. McDonald, Bridgette D. Semple, Mujun Sun, Sandy R. Shultz

**Affiliations:** ^1^Department of Neuroscience, Central Clinical School, Monash University, Melbourne, VIC, Australia; ^2^Division of Infectious Disease and Immune Defense, Walter and Eliza Hall Institute of Medical Research, Parkville, VIC, Australia; ^3^Monash Health Translation Precinct, Monash University, Melbourne, VIC, Australia; ^4^Health Sciences, Vancouver Island University, Nanaimo, BC, Canada

**Keywords:** epileptogenesis, immune response, neuroinflammation, oxidative stress, post-traumatic epilepsy (PTE)

## Abstract

**Introduction:**

Post-traumatic epilepsy (PTE) is a debilitating chronic outcome of traumatic brain injury (TBI), and neuroinflammation is implicated in increased seizure susceptibility and epileptogenesis. However, how common clinical factors, such as infection, may modify neuroinflammation and PTE development has been understudied. The neurotropic parasite, *Toxoplasma gondii (T. gondii)* incurably infects one-third of the world’s population. Thus, many TBI patients have a pre-existing *T. gondii* infection at the time of injury. *T. gondii* infection results in chronic low-grade inflammation and altered signaling pathways within the brain, and preliminary clinical evidence suggest that it may be a risk factor for epilepsy. Despite this, no studies have considered how a pre-existing *T. gondii* infection may alter the development of PTE.

**Methods:**

This study aimed to provide insight into this knowledge gap by assessing how a pre-existing *T. gondii* infection alters susceptibility to, and severity of, pentylenetetrazol (PTZ)-induced seizures (i.e., a surrogate marker of epileptogenesis/PTE) at a chronic stage of TBI recovery. We hypothesized that *T. gondii* will increase the likelihood and severity of seizures following PTZ administration, and that this would occur in the presence of intensified neuroinflammation. To test this, 6-week old male and female C57BL/6 Jax mice were intraperitoneally injected with 50,000 *T. gondii* tachyzoites or with the PBS vehicle only. At 12-weeks old, mice either received a severe TBI *via* controlled cortical impact or sham injury. At 18-weeks post-injury, mice were administered 40 mg/kg PTZ and video-recorded for evaluation of seizure susceptibility. Fresh cortical tissue was then collected for gene expression analyses.

**Results:**

Although no synergistic effects were evident between infection and TBI, chronic *T. gondii* infection alone had robust effects on the PTZ-seizure response and gene expression of markers related to inflammatory, oxidative stress, and glutamatergic pathways. In addition to this, females were more susceptible to PTZ-induced seizures than males. While TBI did not impact PTZ responses, injury effects were evident at the molecular level.

**Discussion:**

Our data suggests that a pre-existing *T. gondii* infection is an important modifier of seizure susceptibility independent of brain injury, and considerable attention should be directed toward delineating the mechanisms underlying this pro-epileptogenic factor.

## 1. Introduction

Traumatic brain injury (TBI) greatly contributes to morbidity and mortality worldwide, and one of the most debilitating long-term consequences of TBI is the development of post-traumatic epilepsy (PTE) (Christensen et al., 2009; GBD 2016 Neurology Collaborators, 2019). PTE is defined as one or more unprovoked seizures that occur at least one week after TBI (Verellen and Cavazos, 2010). PTE accounts for 10–20% of acquired epilepsy in the general population and it is reported that between 4 and 50% of individuals with TBI develop PTE (Degrauw et al., 2018; Tubi et al., 2019; Pease et al., 2022). PTE diagnosis is also associated with heightened morbidity and mortality (Burke et al., 2021; Karlander et al., 2022; Ngadimon et al., 2022).

There is evidence that neuroinflammation can increase seizure susceptibility and the likelihood of PTE development (Webster et al., 2017; Sharma et al., 2019). A role for microglia, astrocytes, chemokines, and cytokines have been described in the progression of PTE (Lu et al., 2021; Radpour et al., 2022). In addition, pre-clinical studies indicate that peripheral inflammation and cerebral infection can alter susceptibility to seizures *via* either a primed immune environment or changes to brain pathophysiology (Ho et al., 2015; Grauncke et al., 2016; Ssentongo et al., 2017; Huang et al., 2018, 2022). However, in the context of PTE, infection has only been considered as a “second-hit” on the back of the neuroinflammatory processes of TBI, rather than as a pre-existing insult or concurrent factor that can modify the neuroinflammatory environment, neural connectivity, and gross anatomy (Sharma et al., 2021; Wang et al., 2021).

*Toxoplasma gondii* (*T. gondii*), which belongs to the Apicomplexa phylum, is estimated to chronically and incurably infect one-third of the population worldwide (Montoya and Liesenfeld, 2004). As such, a significant proportion of TBI patients may have a pre-existing infection at the time of injury. As an obligate intracellular parasite, *T. gondii* invades host cells to survive and replicate, and once in the central nervous system, *T. gondii* can differentiate into an encysted and slow replicating form that is resistant to immune clearance. A large majority of clinical cases of chronic *T. gondii* infection occur with type II strains such as Prugniaud (Pru) or ME49, and chronic infection is characterized by low-grade persistent neuroinflammation (Howe and Sibley, 1995; Bergersen et al., 2021; Egorov et al., 2021). *T. gondii* infection has also been linked to changes in glutamate excitotoxicity and spontaneous seizures in rodent models, and some preliminary clinical findings suggest it as a risk factor for epilepsy (Brooks et al., 2015; David et al., 2016; Sadeghi et al., 2019; Alizadeh Khatir et al., 2021). Yet, no studies to date have investigated *T. gondii* as a modifier of epileptogenesis in the context of TBI.

Therefore, this study aimed to assess how a pre-existing *T. gondii* infection alters susceptibility to, and severity of, pentylenetetrazol (PTZ)-induced seizures (i.e., a surrogate indicator of epileptogenesis/PTE) at a chronic stage of TBI recovery. We hypothesized that combined, *T. gondii* infection and TBI would result in exacerbated seizure susceptibility and severity, and that this would be coupled with an amplified neuroinflammatory environment.

## 2. Materials and methods

### 2.1. Animals

A total of 96 C57BL/6J (Jax) (49 male, 47 female) mice were obtained from the Alfred Medical Research and Education Precinct (AMREP) Animal Services (Melbourne, Australia) for use in this study. Mice were group-housed in ventilated Optimice^®^ cages (3–6 mice/cage/sex) under a 12-h light/dark cycle and were given access to food and water *ad libitum* for the duration of the experiment. All procedures were approved by the AMREP Animal Ethics Committee (#E/2005/2020/M) and performed in accordance with the guidelines of the Australian Code of Practice for the Care and Use of Animals for Scientific Purposes by the Australian National Health and Medical Research Council.

### 2.2. Experimental design

**FIGURE 1:**
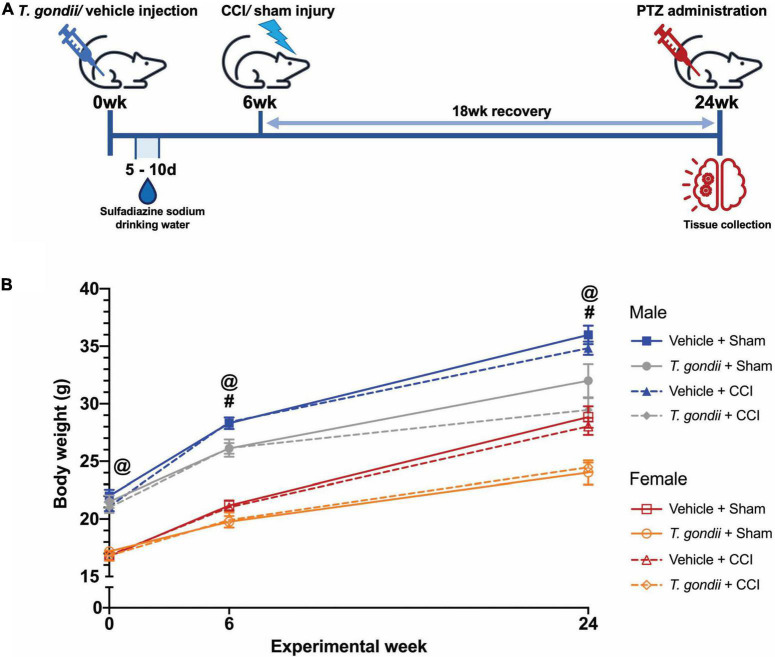
Study timeline. **(A)** All mice were injected with either *Toxoplasma gondii* or vehicle only. Six-weeks were allowed to pass to establish a chronic infection. At this point, mice received either a controlled cortical impact (CCI) or sham injury. After an 18-week recovery (i.e., at 24-weeks from the initial *T. gondii* or vehicle injection), mice underwent pentylenetetrazol (PTZ) administration for assessment of seizure susceptibility immediately prior to euthanasia and tissue collection. **(B)** Body weight significantly differed between male and female mice throughout the study, with males weighing more than females. By 6-weeks post-injection, *T. gondii* mice weighed significantly less than vehicle groups, and this difference was sustained until PTZ administration/endpoint. ^#^*T. gondii*-infected mice significantly differ from vehicle mice, ^@^female mice significantly differ from male mice, *p* < 0.05. *n* = 10–12/group/sex. Data shown is mean ± SEM.

At 6-weeks of age, mice were randomly allocated to receive either a single intraperitoneal (i.p.) injection of *T. gondii* tachyzoites or vehicle only (Figure 1A). All mice received 100 μg/mL sulfadiazine sodium (Sigma Aldrich, Burlington, MA, USA) in their drinking water on days 5 to 10 after injection to aid recovery from the acute stage of infection. This treatment is commonly used to control tachyzoite proliferation during active toxoplasmosis and reduce animal death throughout the acute stage of infection (Ochiai et al., 2015; Zhou et al., 2019). Of 48 mice (25 male, 23 female) infected with *T. gondii*, 2 (1 male, 1 female) did not recover from the acute stage of infection (4.17% total mortality). Mice were allowed 6-weeks after injection for a chronic *T. gondii* infection to establish, and at this point, received either a TBI that was induced *via* the controlled cortical impact (CCI) model or a sham injury. Therefore, this study was comprised of four experimental groups per sex: Vehicle + Sham; *T. gondii* + Sham; Vehicle + CCI; and *T. gondii* + CCI. Some mice (2 *T. gondii* + CCI males, 1 *T. gondii* + Sham female) died prematurely to the 18-week post-injury endpoint. As such, 91 mice underwent PTZ injection and endpoint at 18-weeks post-injury. Investigators were blinded to group allocations immediately following injury throughout all experimentation and data analyses. Body weights were monitored throughout the study (Figure 1B). Males weighed more than females throughout the study. By 6-weeks post-injection, *T. gondii* mice weighed significantly less than vehicle groups, and this difference was sustained until PTZ administration/endpoint.

### 2.3. Chronic *Toxoplasma gondii* infection

*Toxoplasma gondii* (Pru:tdTomato) tachyzoites were maintained by passage on human foreskin fibroblasts and resuspended in Dulbecco’s phosphate buffered saline (DPBS) to a concentration of 50,000 *T. gondii* tachyzoites per 200 μL DPBS (Tyebji et al., 2019). Mice allocated to *T. gondii* groups subsequently received a single i.p., injection of 50,000 tachyzoites and were monitored across a 6-week period for sickness behaviors as previously described (Tyebji et al., 2019). Mice allocated to vehicle groups received 200 μL DPBS only and were similarly monitored across a 6-week period.

### 2.4. Controlled cortical impact

The CCI model in mice reproduces several pathophysiological and functional features commonly seen in TBI patients (Xiong et al., 2013). CCI was performed at 6-weeks post-injection (12-weeks old) as previously described (Webster et al., 2019). Anesthesia was induced by 4% isoflurane in 1 L/min oxygen and maintained for surgery at 1.5–2% isoflurane *via* nose cone. Briefly, mice were stabilized in a stereotaxic frame and a midline incision was made to expose the skull. A ∼3 mm diameter craniotomy above the left parietal lobe was performed with a microdrill (0.6 mm drill bit) at a position of 1 mm posterior to Bregma, 1 mm lateral to the midline-sagittal suture, and 1 mm anterior to Lambda. Severe injury parameters were set using an electronic CCI device (Custom Design and Fabrication Inc., Sandston, VA, USA) at 4.5 m/s velocity, 1.71 mm depth, and 150 ms dwell time. Sham injuries were performed as described above, without the delivery of an impact to the parietal lobe. All surgical tools and the impactor tip were cleaned with 80% ethanol between animals and tools were additionally sterilized using a hot bead sterilizer.

### 2.5. Pentylenetetrazol seizure induction and seizure susceptibility analysis

As a non-competitive γ-aminobutyric acid (GABA)_*A*_ receptor antagonist, PTZ is able to close chloride channels and prevent hyperpolarization, leading to continuous stimulation of cortical neurons and convulsions (Viswanatha et al., 2020). Prior to euthanasia at 18-weeks post-injury, mice were administered 40 mg/kg PTZ i.p. (Sigma Aldrich) and video-recorded for a period of up to 30 min for evaluation of seizure susceptibility, using a modified seizure severity score to assess behavioral responses (Table 1; Van Erum et al., 2019; Sharma et al., 2021). This dose was chosen based on previous work by Sharma et al. (2019) and Van Erum et al. (2019), which demonstrated a wide range of behavioral responses at 40 mg/kg PTZ i.p., and on average mice reach a score of 4. Briefly, responses assessed ranged from normal behavior (score 0) to tonic extension and death (score 7), with scores of 4–7 being considered as generalized convulsive seizures. As such, seizure latency was considered as the time taken to reach a score greater than or equal to 4. Seizure duration was considered as the total time in which a mouse exhibited scores 4 to 6, and if a score of 7 was reached (i.e., premature death), a maximum value of 1,800 s was assigned.

**TABLE 1:** Modified seizure severity score.

Seizure severity score	Description
0	Normal behavior
1	Hypomobility, behavioral arrest, loss of posture, or immobility
2	Isolated and focal myoclonic jerks including facial twitches
3	Strong myoclonic jerks including head nodding and unilateral forelimb jerks
4	Clonic seizure involving movements such as forelimb and facial clonus, with or without rearing
5	Tonic-clonic seizure involving clonus of all limbs while maintaining balance
6	Tonic-clonic seizure with loss of righting reflex (lying on side), and may include wild running and jumping
7	Tonic extension, possibly leading to respiratory arrest, and death

### 2.6. Tissue collection

Mice were euthanized at 18-weeks post-surgery immediately following PTZ challenge (i.e., at a maximum of 30 min from PTZ administration), with a single i.p., injection of sodium pentobarbitone (80 mg/kg; Lethabarb; Virbac, Australia). Next, fresh brain tissue from the ipsilateral parietal cortex was collected for gene expression analysis. All samples were frozen on dry ice and stored at −80°C prior to analysis.

In addition, a subset of mice underwent transcardial perfusion with ice-cold sterile saline (0.9% NaCl w/v) followed by 4% paraformaldehyde (PFA) to fix the brain tissue for confirmation of brain damage due to CCI *via* structural MRI. Brains were post-fixed in 4% PFA overnight at 4°C, washed twice in 1x PBS, then transferred to 1x PBS for storage at 4°C prior to analysis.

### 2.7. Gene expression analysis

Gene expression analysis was used to examine genes related to immune, oxidative stress, glutamate, and apoptotic pathways. Total RNA was isolated by hand from 20 mg of ipsilateral parietal cortex tissue using a RNeasy^®^ Mini Kit (Qiagen, Germantown, MD, USA). A total of 200 ng of yielded RNA proceeded to cDNA synthesis using Quantabio qScript XLT cDNA SuperMix (Quantabio). Multiplex qPCR was performed with Fluidigm BioMark™ HD. For each sample, 1.25 μL of the resulting cDNA was combined with 3.75 μL of Sample Pre Mix (Life Technologies TaqMan^®^ PreAmp Master Mix and Pooled Taqman assays) and pre-amplified for 14 cycles. The reaction products were diluted 1:5 and loaded onto the Gene expression IFC according to Fluidigm^®^ IFC Standard Taqman Gene expression workflow. 37 TaqMan^®^ gene expression assays related to immune cells, neuroinflammation, oxidative stress, apoptosis, and the glutamate pathway, and 4 housekeeping gene assays were used as detailed in Table 2. Cycle threshold (Ct) values were collected for analysis, using the 2^–Δ^ ^Δ^ ^Ct^ method.

**TABLE 2:** Immune cell, neuroinflammatory, glutamate pathway, and housekeeping genes analyzed *via* RT-PCR using Taqman assays.

	Gene name	Taqman assay ID		Gene name	Taqman assay ID
Housekeeping			Neuroinflammation		
1	*YWHAZ*	Mm01722325_m1	21	*TSPO*	Mm00437828_m1
2	*ACTB*	Mm00607939_s1	22	*NLRP3*	Mm00840904_m1
3	*GAPDH*	Mm99999915_g1	23	*IL1β*	Mm00434228_m1
4	*UBC*	Mm02525934_g1	24	*TNFα*	Mm99999068_m1
Immune cell			25	*CSF1/M-CSF*	Mm00432688_m1
5	*GFAP*	Mm01253033_m1	26	*CSF2/GM-CF*	Mm00438328_m1
6	*CD45*	Mm01293577_m1	27	*IFNγ*	Mm00801778_m1
7	*CD86*	Mm00444543_m1	28	*ARG1*	Mm00475988_m1
8	*CD206*	Mm01329362_m1	29	*IL17A*	Mm00439618_m1
9	*IBA1*	Mm00479862_g1	30	*IL33*	Mm00505403_m1
10	*TMEM119*	Mm00525305_m1	Oxidative stress		
11	*TREM2*	Mm04209424_g1	31	*IDO1*	Mm00492586_m1
12	*CCL2*	Mm00441242_m1	32	*NOS2*	Mm00440502_m1
13	*CXCR3*	Mm00438259_m1	33	*CYBB*	Mm01287743_m1
14	*GATA3*	Mm00484683_m1	Apoptosis		
15	*SOCS1*	Mm00782550_s1	34	*APAF1*	Mm01223702_m1
16	*STAT1*	Mm01257286_m1	35	*CASP9*	Mm00516563_m1
17	*CD62L/SELL*	Mm00441291_m1	36	*CASP3*	Mm01195085_m1
18	*KLRG1*	Mm00516879_m1	Glutamate pathway		
19	*FOXP3*	Mm00475162_m1	37	*GLT1*	Mm01275814_m1
20	*PRDM1*	Mm00476128_m1	38	*GLAST*	Mm01306917_g1
			39	*GAD1*	Mm04207432_g1
			40	*PHGDH*	Mm01623589_g1
			41	*GLUL*	Mm00725701_s1

### 2.8. *Ex vivo* MRI

**FIGURE 2:**
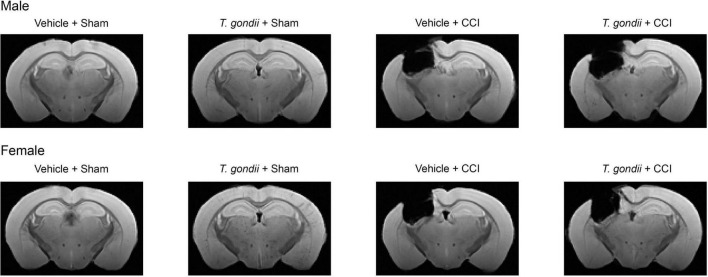
Controlled cortical impact (CCI) results in long-term structural brain damage. Template structural MRI images show chronic brain damage in CCI groups compared to sham groups.

To verify that the CCI resulted in brain damage, structural MRI data was performed on a subset of brains that were not used for gene expression analysis (Male Vehicle + Sham = 7; Male *T. gondii* + Sham = 7; Male Vehicle + CCI = 7; Male *T. gondii* + CCI = 6; Female Vehicle + Sham = 7; Female *T. gondii* + Sham = 6; Female Vehicle + CCI = 7; Female *T. gondii* + CCI = 6). Fixed brains were washed in PBS and positioned in fomblin (Solvay Solexis, USA) for imaging with a 9.4T Bruker MRI. A 3D multi-gradient echo image was acquired to confirm injury. Imaging parameters included: repetition time = 75 ms; echo times = 5, 10, 15, …, 50 ms; field of view = 16.32 × 10.88 × 7.14 mm^3^; matrix size = 192 × 128 × 84; and resolution = 85 × 85 × 85 μm^3^. Images were reconstructed using in-house code written in MATLAB (r2021a, MathWorks, Natick, MA, USA) and templates constructed for each group using the mean echo image as described previously (Wright et al., 2018, 2019). As shown in Figure 2, the CCI groups had substantial structural brain damage relative to the sham groups.

### 2.9. Statistical analysis

Data was analyzed with SPSS 28.0 software (IBM Corp., Armonk, USA). Responses to PTZ administration and gene expression outcomes were analyzed by 3-way ANOVA, with sex, infection, and injury as between-subject factors, except for the percentage of mice that exhibited a generalized convulsive seizure which was analyzed using Fisher’s exact test. Bonferroni *post-hoc* comparisons were carried out when appropriate. Statistical significance was set as *p* < 0.05.

## 3. Results

### 3.1. *Toxoplasma gondii* infection increases chronic susceptibility to, and severity of, PTZ-induced seizures

**FIGURE 3:**
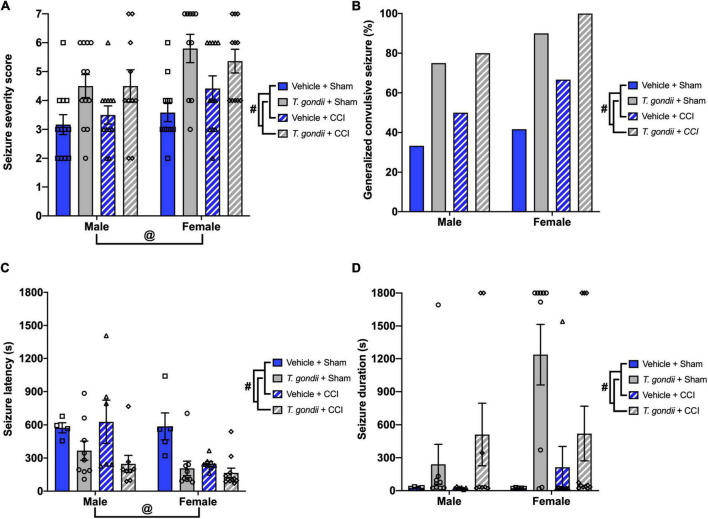
*Toxoplasma gondii* infection increased chronic susceptibility to pentylenetetrazol (PTZ)-induced seizures irrespective of injury. **(A)** *T. gondii* and female mice reached higher seizure severity scores compared to vehicle and male groups, respectively. **(B)** Overall, a higher percentage of mice in *T. gondii* groups developed generalized convulsive seizures compared to vehicle groups, yet there was no difference between injured and sham groups. **(C)** Of the mice that exhibited a generalized convulsive seizure, female and *T. gondii* groups had decreased seizure latency. **(D)** In addition, *T. gondii*-infected mice had increased seizure duration compared to vehicle mice. ^#^*T. gondii*-infected mice significantly differ from vehicle mice, ^@^female mice significantly differ from male mice, *p* < 0.05. *n* = 10–12/group/sex. Data shown is mean ± SEM.

Following PTZ injection, a main effect of infection [*F*_(1,83)_ = 22.70, *p* < 0.001; Figure 3A] and sex [*F*_(1,83)_ = 9.19, *p* = 0.003; Figure 3A] was observed on the modified seizure severity score, indicating that both *T. gondii*-infected and female mice reached higher scores than vehicle and male mice, respectively. Additionally, a higher proportion of mice in *T. gondii* groups developed generalized convulsive seizures compared to mice in vehicle groups (*p* < 0.001; Figure 3B). No statistically significant effect of injury or sex was observed on this measure. Of mice that developed a generalized convulsive seizure, a main effect of infection [*F*_(1,52)_ = 16.98, *p* < 0.001; Figure 3C] and sex [*F*_(1,52)_ = 5.71, *p* = 0.021; Figure 3C] was evident on seizure latency, with *T. gondii*-infected and female mice found to take less time to exhibit a generalized convulsive seizure compared to vehicle and male mice, respectively. Further to this, *T. gondii*-infected mice displayed increased seizure duration compared to vehicle mice [*F*_(1,52)_ = 10.26, *p* = 0.002; Figure 3D].

### 3.2. *Toxoplasma gondii* infection chronically alters leukocyte and glial gene expression in the ipsilateral cortex

**FIGURE 4:**
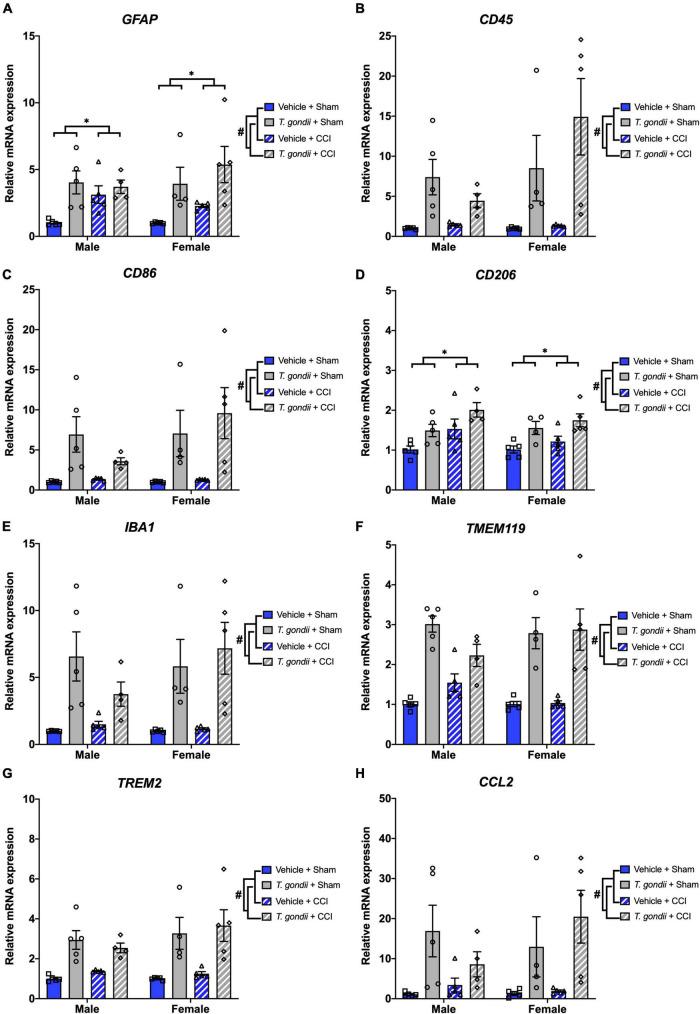
*Toxoplasma gondii* infection increased leukocyte and glial cell gene expression in the ipsilateral cortex of mice chronically post-controlled cortical impact (CCI). Gene expression of *GFAP* **(A)**, *CD45* **(B)**, *CD86* **(C)**, *CD206* **(D)**, *IBA1* **(E)**, *TMEM119* **(F)**, *TREM2* **(G)**, and *CCL2* **(H)** was increased in *T. gondii* infected mice compared to vehicle mice. Gene expression of *GFAP* **(A)** and *CD206* **(D)** was also increased in mice that received a CCI compared to sham-injured mice. ^#^*T. gondii*-infected mice significantly differ from vehicle mice, *CCI mice significantly differ from sham-injured mice, *p* < 0.05. *n* = 4–5/group/sex. Data shown is mean ± SEM.

Genes related to leukocytes and glial cells were assessed using Multiplex qPCR. There was a significant effect of infection on the mRNA expression of *GFAP* [*F*_(1,30)_ = 19.98, *p* < 0.001; Figure 4A], *CD45* [*F*_(1,30)_ = 21.44, *p* < 0.001; Figure 4B], *CD86* [*F*_(1,30)_ = 22.14, *p* < 0.001; Figure 4C], *CD206* [*F*_(1,30)_ = 20.10, *p* < 0.001; Figure 4D], *IBA1* [*F*_(1,30)_ = 30.28, *p* < 0.001; Figure 4E], *TMEM119* [*F*_(1,30)_ = 69.42, *p* < 0.001; Figure 4F], *TREM2* [*F*_(1,30)_ = 41.52, *p* < 0.001; Figure 4G], and *CCL2* [*F*_(1,30)_ = 18.06, *p* < 0.001; Figure 4H], such that expression was increased in *T. gondii*-infected mice compared to vehicle mice. There was also a main effect of injury on the expression of *GFAP* [*F*_(1,30)_ = 4.33, *p* = 0.046; Figure 4A] and *CD206* [*F*_(1,30)_ = 9.91, *p* = 0.004; Figure 4D], with expression significantly higher in injured mice compared to sham-injured mice.

**FIGURE 5:**
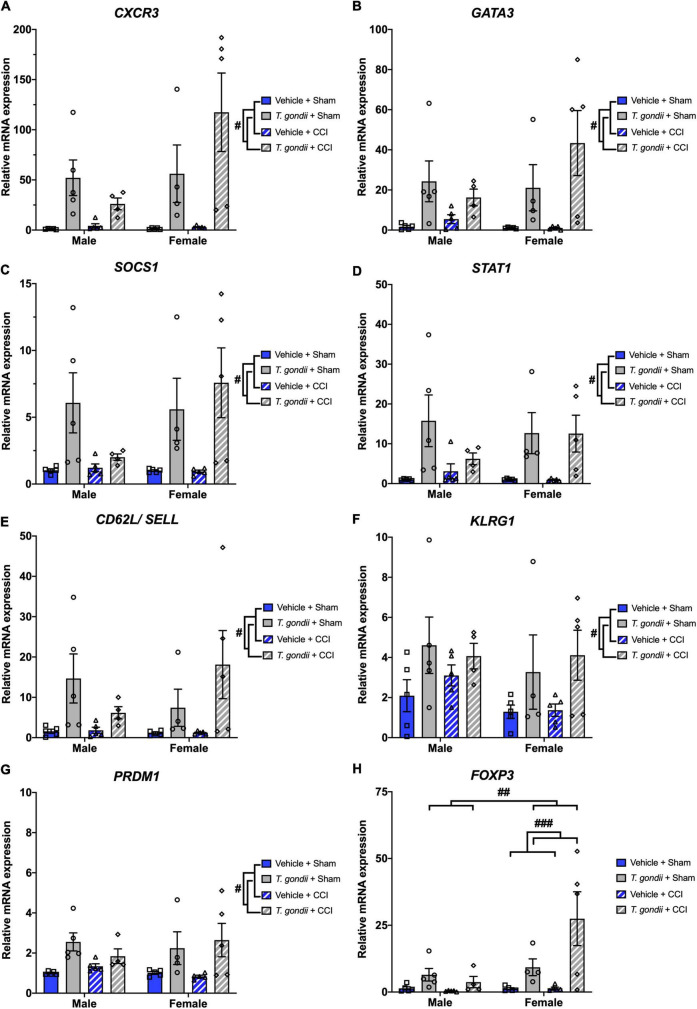
*Toxoplasma gondii* infection increases gene expression of CD4^+^ and CD8^+^ T-cell markers within the ipsilateral cortex chronically post-controlled cortical impact (CCI). *T. gondii*-infected mice had increased *CXCR3* **(A)**, *GATA3* **(B)**, *SOCS1* **(C)**, *STAT1* **(D)**, *CD62L/SELL* **(E)**, *KLRG1* **(F)**, *PRDM1* **(G)**, and *FOXP3* **(H)**. On top of this, *T. gondii*-infected females had increased expression of FOXP3 compared to both *T. gondii*-infected males and vehicle females. ^#^*T. gondii*-infected mice significantly differ from vehicle mice, ^##^*T. gondii*-infected females significantly differ from *T. gondii*-infected males, ^###^*T. gondii*-infected females significantly differ from vehicle females, *p* < 0.05. *n* = 4–5/group/sex. Data shown is mean ± SEM.

Gene expression of various CD4^+^ and CD8^+^ T-cell markers were also assessed using Multiplex qPCR. A main effect of infection was evident on the expression of *CXCR3* [*F*_(1,30)_ = 21.90, *p* < 0.001; Figure 5A], *GATA3* [*F*_(1,30)_ = 17.52, *p* < 0.001; Figure 5B], *SOCS1* [*F*_(1,30)_ = 16.84, *p* < 0.001; Figure 5C], *STAT1* [*F*_(1,30)_ = 17.72, *p* < 0.001; Figure 5D], *CD62L/SELL* [*F*_(1,30)_ = 11.80, *p* = 0.002; Figure 5E], *KLRG1* [*F*_(1,30)_ = 8.53, *p* = 0.007; Figure 5F], and *PRDM1* [*F*_(1,30)_ = 16.35, *p* < 0.001; Figure 5G], by which all were increased in *T. gondii*-infected mice compared to vehicle mice. Main effects of sex and infection, as well as a sex by infection interaction, were noted for the expression of *FOXP3* [*F*_(1,30)_ = 5.69, *p* = 0.024, *F*_(1,30)_ = 13.69, *p* < 0.001, and *F*_(1,30)_ = 5.01, *p* = 0.033, respectively; Figure 5H], with *T. gondii*-infected females having significantly increased *FOXP3* expression compared to males (*p* = 0.003) and compared to vehicle females (*p* < 0.001).

### 3.3. *Toxoplasma gondii* infection chronically amplifies neuroinflammatory and oxidative stress mediators

**FIGURE 6:**
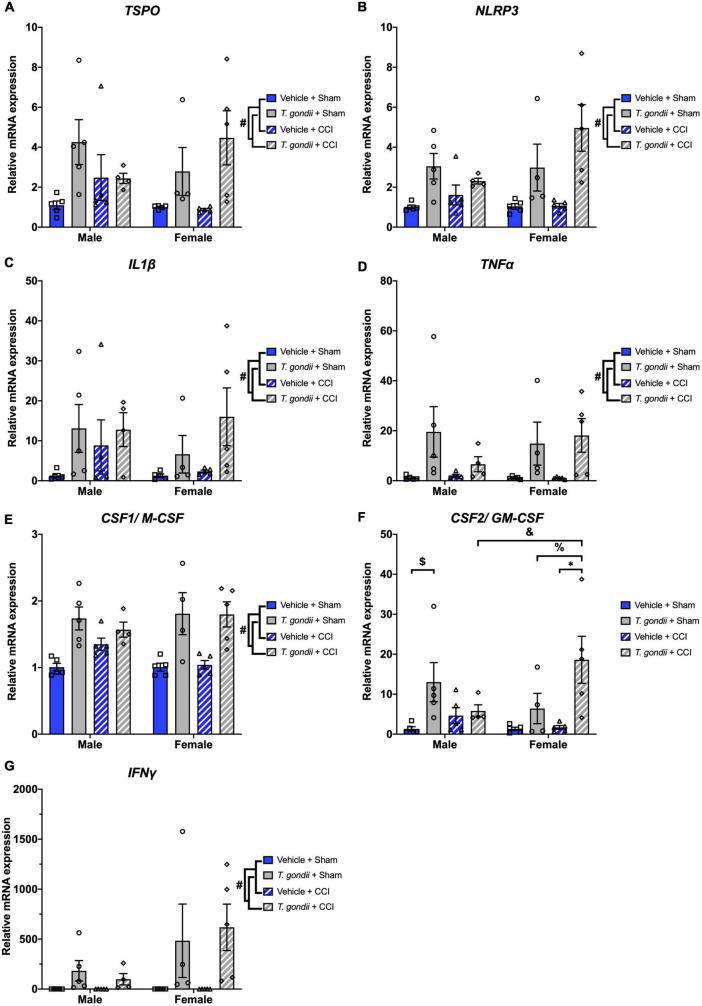
Chronic *Toxoplasma gondii* infection increases gene expression of neuroinflammatory mediators in both sexes. Increased expression of *TSPO* **(A)**, *NLRP3* **(B)**, *IL1*β **(C)**, *TNFα* **(D)**, *CSF1/M-CSF* **(E)**, *CSF2/GM-CSF* **(F)**, and *IFNγ* **(G)** was increased in *T. gondii*-infected mice compared to vehicle mice. Further to this, *T. gondii* + controlled cortical impact (CCI) females also had increased expression of *CSF2/GM-CSF* compared to *T. gondii* + CCI males, *T. gondii* + sham females, and vehicle + CCI females. *T. gondii* + sham males also had increased expression of *CSF2/GM-CSF* compared to vehicle + sham males. ^#^*T. gondii*-infected mice significantly differ from vehicle mice, ^$^*T. gondii* + sham males significantly differ from vehicle + sham males, ^&^*T. gondii* + CCI males significantly differ from *T. gondii* + CCI females, % *T. gondii* + sham females significantly differ from *T. gondii* + CCI females, *vehicle + CCI females significantly differ from *T. gondii* + CCI females, *p* < 0.05. *n* = 4–5/group/sex. Data shown is mean ± SEM.

Next, gene expression of neuroinflammatory and oxidative stress mediators was investigated. A main effect of infection was found on the expression of *TSPO* [*F*_(1,30)_ = 11.88, *p* = 0.002; Figure 6A], *NLRP3* [*F*_(1,30)_ = 22.37, *p* < 0.001; Figure 6B], *IL1*β [*F*_(1,30)_ = 6.94, *p* = 0.013; Figure 6C], *TNFα* [*F*_(1,30)_ = 12.88, *p* = 0.001; Figure 6D], *CSF1/M-CSF* [*F*_(1,30)_ = 36.43, *p* < 0.001; Figure 6E], *CSF2/GM-CSF* [*F*_(1,30)_ = 14.66, *p* < 0.001; Figure 6F], and *IFNγ* [*F*_(1,30)_ = 11.36, *p* = 0.002; Figure 6G], where for all these genes *T. gondii*-infected mice exhibited increased expression compared to vehicle mice. A sex by infection by injury interaction was detected for the expression of *CSF2/GM-CSF* [*F*_(1,30)_ = 6.03, *p* = 0.020]. *Post-hoc* analyses revealed that *T. gondii* + CCI females had increased expression compared to *T. gondii* + CCI males (*p* = 0.010), *T. gondii* + sham females (*p* = 0.014), and vehicle + CCI females (*p* < 0.001), and *T. gondii* + sham males had increased expression compared to vehicle + sham males (*p* = 0.013).

**FIGURE 7:**
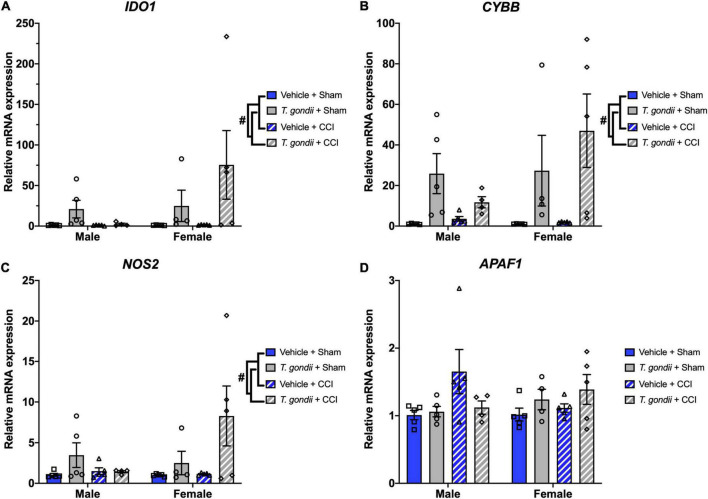
Chronic *Toxoplasma gondii* infection increases gene expression of oxidative stress mediators. Gene expression of *IDO1* **(A)**, *CYBB* **(B)**, and *NOS2* **(C)** was increased in *T. gondii*-infected mice compared to vehicle mice. A sex by infection interaction was detected for the expression of *APAF1* **(D)**, however, *post-hoc* analyses failed to reach significance. ^#^*T. gondii*-infected mice significantly differ from vehicle mice, *p* < 0.05. *n* = 4–5/group/sex. Data shown is mean ± SEM.

A main effect of infection was also found on the expression of *IDO1* [*F*_(1,30)_ = 5.86, *p* = 0.022; Figure 7A], *CYBB* [*F*_(1,30)_ = 15.67, *p* < 0.001; Figure 7B], and *NOS2* [*F*_(1,30)_ = 6.16, *p* = 0.019; Figure 7C], where *T. gondii*-infected mice had increased expression compared to vehicle mice. A sex by infection interaction was detected for the expression of *APAF1* [*F*_(1,30)_ = 4.24, *p* = 0.048; Figure 7D] although *post-hoc* analyses failed to reach significance.

### 3.4. *Toxoplasma gondii* infection, CCI, and sex chronically alter expression of glutamate pathway genes in the ipsilateral cortex

**FIGURE 8:**
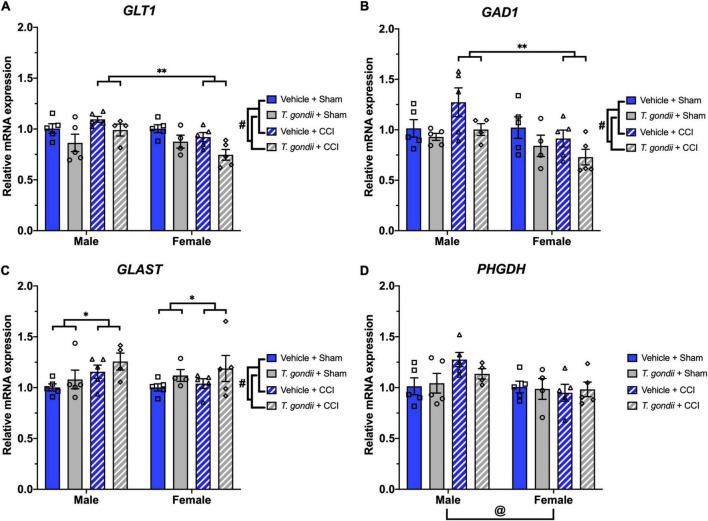
*Toxoplasma gondii*, controlled cortical impact (CCI), and sex alter the expression of glutamate pathway components. Expression of *GLT1* **(A)** and *GAD1* **(B)** were decreased in *T. gondii*-infected and female mice compared to vehicle and male mice, respectively. CCI females had lower expression of *GLT1* and *GAD1* compared to CCI males. In contrast, *T. gondii*-infected and CCI mice had increased expression of *GLAST* **(C)** compared to vehicle and sham mice, respectively. Expression levels of *PHGDH* **(D)** were lower in female mice compared to males. ^#^*T. gondii*-infected mice significantly differ from vehicle mice, *sham mice significantly differ from CCI mice, ^@^male mice significantly differ from female mice, ^**^male CCI mice significantly differ from female CCI mice, *p* < 0.05. *n* = 4–5/group/sex. Data shown is mean ± SEM.

Main effects of infection and sex, as well as a sex by injury interaction, was evident on expression levels of *GLT1* [*F*_(1,30)_ = 12.55, *p* = 0.001, *F*_(1,30)_ = 6.90, *p* = 0.013, and *F*_(1,30)_ = 7.68, *p* = 0.010, respectively; Figure 8A] and *GAD1* [*F*_(1,30)_ = 7.35, *p* = 0.011, *F*_(1,30)_ = 7.23, *p* = 0.012, and *F*_(1,30)_ = 4.32, *p* = 0.046, respectively; Figure 8B], with *post-hoc* analyses revealing CCI females had lower expression levels of *GLT1* (*p* < 0.001) and *GAD1* (*p* = 0.002) compared to CCI males. Main effects of infection and injury were seen on *GLAST* expression [*F*_(1,30)_ = 4.35, *p* = 0.046 and *F*_(1,30)_ = 4.20, *p* = 0.049, respectively; Figure 8C] in which *T. gondii* and CCI groups had elevated expression levels compared to vehicle and sham groups, respectively. A main effect of sex was found on expression of *PHGDH* [*F*_(1,30)_ = 5.84, *p* = 0.022; Figure 8D] such that females had lower expression of *PHGDH* compared to males.

No significant effects of sex, infection or injury were found on expression of *ARG1*, *IL17A*, *IL33*, *CASP9*, *CASP3*, *GLUL*, *YWHAZ*, *ACTB*, *GAPDH*, and *UBC* (data not shown).

## 4. Discussion

This study examined the effects of a pre-existing *T. gondii* infection on susceptibility to, and severity of, PTZ-induced seizures at the chronic stage of TBI recovery. We hypothesized that in a combined chronic *T. gondii* infection and TBI setting, seizure severity, and susceptibility would be exacerbated, and *T. gondii* + CCI mice would have an exacerbated neuroinflammatory environment compared to other groups. Contrary to our hypotheses, no synergistic effects between infection and TBI were found. However, chronic *T. gondii* infection alone had robust effects on the PTZ-seizure response and gene expression of markers related to inflammatory, oxidative stress, and glutamatergic pathways. In addition to this, females were more susceptible than males to PTZ administration. Although, TBI did not influence PTZ outcomes, injury effects were evident at the structural and molecular level.

Through assessment of susceptibility to PTZ as a surrogate indicator of epileptogenesis, we found that seizure severity and duration, as well as the percentage of each group that developed a generalized convulsive seizure, were exacerbated for *T. gondii*-infected mice compared to the non-infected mice, irrespective of injury type. In addition, *T. gondii*-infected mice had decreased seizure latency in contrast to non-infected mice. These findings align with a previous study that reported increased seizure scores and decreased seizure latency after a PTZ challenge in C57BL/6 mice infected with a type II *T. gondii* strain (ME49) (Brooks et al., 2015). However, the current study is the first to our knowledge to examine not only seizure susceptibility/severity in *T. gondii*-infected male and female mice, but also to assess these measures at a chronic timepoint after injection, and in conjunction with quantification of a large range of genes associated with several neuroimmune pathways.

Interestingly, female mice took less time to develop a generalized convulsive seizure and had a higher seizure severity when compared to males, contrary to previous reports that found no difference between sexes (Yuskaitis et al., 2021). This may be due to the use of different strains, age and physiologic stressors between studies. Although no statistically significant sex by infection interactions were found on PTZ outcomes, it should be noted that the *T. gondii* infection paradigm did not control for body weight (i.e., all mice received a single i.p., injection of 50,000 tachyzoites). Therefore, because the females weighed less than males they were exposed to a higher *T. gondii* dose based on body weight. In contrast to previous findings (Semple et al., 2017; Sharma et al., 2021), we found no effect of TBI on response to PTZ at this time point. This may be explained by differences in the age at time of injury (i.e., pediatric compared to adult) as pediatric populations respond differently to TBI, have a higher incidence of PTE, and are more likely to develop post-traumatic seizures compared to older groups (Hwang et al., 2019; Webster et al., 2019; Elsamadicy et al., 2021; Mariajoseph et al., 2022). It may also be explained by differences in strain and methodologies used, as murine strains present diverse responses to PTZ, responses can vary between injury models, lower PTZ dosage can result in a greater proportion of subconvulsive events, epileptogenic sequelae vary with the stage of recovery being assessed, and physiologic stressors including EEG implantation and single-housing can increase the number of PTZ-induced seizures (Bolkvadze and Pitkänen, 2012; Mukherjee et al., 2013; Smith et al., 2018; Yuskaitis et al., 2021). Furthermore, although no effect of TBI on response to PTZ was evident at this timepoint, it is possible that the CCI was still pro-epileptogenic in terms of the development of spontaneous seizures. Related to this, although this study demonstrated differences in seizure susceptibility in *T. gondii*-infected mice, interpretation of generalized convulsive seizure responses to a PTZ challenge is limited in its translation to PTE development, and future studies should incorporate EEG-based analysis to accurately assess chronic spontaneous seizures without the use of PTZ.

At the molecular level, *T. gondii* infection was found to alter the expression of a myriad of leukocyte, glial, neuroinflammatory, oxidative stress, and glutamate related genes in the ipsilateral cortex in both males and females. These findings are in line with the current understanding of how a chronic *T. gondii* infection is maintained *via* activation of the host immune system (Wang et al., 2019; Bergersen et al., 2021), although no study to date has depicted a holistic picture of gene expression changes regarding the murine neuroinflammatory environment beyond 8-weeks post-infection. Nonetheless, the overall amplified neuroinflammation in *T. gondii*-infected mice compared to vehicle mice may explain the increased seizure susceptibility in these groups (Deng et al., 2021; Radpour et al., 2022). Expression of *FOXP3* and *CSF2/GM-CSF* was particularly amplified in *T. gondii* + CCI females compared to other groups. The roles of both *FOXP3* and *CSF2/GM-CSF* in epileptogenesis are largely unknown; however, *in situ* work has demonstrated a role for *CSF2/GM-CSF* in disrupted neuronal networks, and patient studies indicate increased frequency of *FOXP3*^+^ cells are positively correlated with age at onset of temporal lobe epilepsy (Vieira et al., 2016; Dikmen et al., 2020). These may therefore contribute to 100% of the *T. gondii* + CCI females developing a generalized convulsive seizure after PTZ injection, although more work will need to be conducted to delineate the mechanistic processes involved. Pertinent to this point, a thorough histopathological investigation for *T. gondii* tachyzoite replication (i.e., a reactivated chronic infection) and resultant structural damage should be conducted given that *T. gondii* tachyzoite replication in the clinical setting can result in encephalitis, ventriculitis, lesions, and seizures (Pradhan et al., 2007; Derouin and Pelloux, 2008). Some murine strains such as C57BL/6 mice, are more susceptible to toxoplasmic encephalitis (Suzuki et al., 1991), and changes including ventricular dilatation have also been reported in the more resistant Swiss-Webster mice after one-year of infection with a type II *T. gondii* strain (Hermes et al., 2008). However, as reactivation of a chronic *T. gondii* infection is also associated with downregulated IFNγ and TNFα expression, and simultaneous depletion of CD4+ and CDD8+ T lymphocytes (Gazzinelli et al., 1992, 1993), among other mechanisms, gene expression analyses in this study do not indicate reactivation of the chronic *T. gondii* infection yet this cannot be confirmed without histopathological investigation. CCI effects were also evident at the molecular level including increased expression of *GFAP*, *CD206*, and *GLAST* in CCI mice compared to sham-injured mice, and increased expression of *GLT1* and *GAD1* in CCI males compared to CCI females. Although a sex-specific finding for *GLT1* and *GAD1* in the context of TBI has not previously been reported, this decrease in CCI females compared to CCI males may further explain the increased susceptibility of females to PTZ injection in this study, given that GLT1 translation has previously been reported to be neuroprotective in the context of pilocarpine-induced temporal lobe epilepsy (Kong et al., 2014).

Although mice were euthanized immediately following PTZ challenge to minimize gene expression changes resulting from PTZ administration and seizure activity, the interpretation of these results is limited without the assessment of gene expression in mice that did not receive PTZ. This is important since subtle gene expression changes of inflammatory mediators in the brain have been demonstrated within 3 h of high-dose PTZ (e.g., 70 and 95 mg/kg); however, lower doses of PTZ similar to what was used in this study do not appear to influence gene expression acutely after exposure (e.g., 30 mg/kg; Amada et al., 2013; Karan et al., 2021). Further to this point, it is possible that *T. gondii* cyst burden and distribution throughout the brain may be meaningful in the explanation of our findings, yet *T. gondii* burden and distribution was not directly assessed in the current study.

In conclusion, the findings of this study demonstrate a robust effect of chronic *T. gondii* infection on not only seizure severity and susceptibility at the chronic stage of recovery, but also on the neuroinflammatory factors that may contribute to the epileptogenic process. Although this study is not without limitations, the findings demonstrate that a pre-existing *T. gondii* infection is a significant modifier of PTZ-induced seizures independent of brain injury, and further attention should be directed toward understanding the mechanisms and potential interventions.

## Data availability statement

The original contributions presented in this study are included in the article/supplementary material, further inquiries can be directed to the corresponding author.

## Ethics statement

The animal study was reviewed and approved by AMREP Animal Ethics Committee.

## Author contributions

SS, MS, and TB conceptualized and designed the study. DW completed MRI analysis. TB and MS completed pentylenetetrazol (PTZ) data collection. TB and SS conducted the data analysis. AV and TW completed gene expression experiments. CT and AU provided the *Toxoplasma gondii* and assisted in establishing the *T. gondii* model. BS assisted with the brain injury and PTZ models. All authors contributed to the article and approved the submitted version.
